# Double-Step Paradigm in Microgravity: Preservation of Sensorimotor Flexibility in Altered Gravitational Force Field

**DOI:** 10.3389/fphys.2020.00377

**Published:** 2020-04-24

**Authors:** L. Bringoux, T. Macaluso, P. Sainton, L. Chomienne, F. Buloup, L. Mouchnino, M. Simoneau, J. Blouin

**Affiliations:** ^1^Aix Marseille Univ, CNRS, ISM, Marseille, France; ^2^Aix Marseille Univ, CNRS, LNC, Marseille, France; ^3^Département de Kinésiologie, Faculté de Médecine, Université Laval, Quebec, QC, Canada; ^4^Centre Interdisciplinaire de Recherche en Réadaptation et Intégration Sociale (CIRRIS) du CIUSSS de la Capitale Nationale, Quebec, QC, Canada

**Keywords:** reaching, pointing, arm kinematics, sensorimotor adaptation, double-step paradigm, microgravity, parabolic flight, weightlessness

## Abstract

The way we can correct our ongoing movements to sudden and unforeseen perturbations is key to our ability to rapidly adjust our behavior to novel environmental demands. Referred to as sensorimotor flexibility, this ability can be assessed by the double-step paradigm in which participants must correct their ongoing arm movements to reach targets that unexpectedly change location (i.e., target jump). While this type of corrections has been demonstrated in normogravity in the extent of reasonable spatiotemporal constraints underpinning the target jumps, less is known about sensorimotor flexibility in altered gravitational force fields. We thus aimed to assess sensorimotor flexibility by comparing online arm pointing corrections observed during microgravity episodes of parabolic flights with normogravity standards. Seven participants were asked to point as fast and as accurately as possible toward one of two visual targets with their right index finger. The targets were aligned vertically in the mid-sagittal plane and were separated by 10 cm. In 20% of the trials, the initially illuminated lower target was switched off at movement onset while the upper target was concomitantly switched on prompting participants to change the trajectory of their ongoing movements. Results showed that, both in normogravity and microgravity, participants successfully performed the pointing task including when the target jumped unexpectedly (i.e., comparable success rate). Most importantly, no significant difference was found in target jump trials regarding arm kinematics between both gravitational environments, neither in terms of peak velocity, relative deceleration duration, peak acceleration or time to peak acceleration. Using inverse dynamics based on experimental and anthropometrical data, we demonstrated that the shoulder torques for accelerating and decelerating the vertical arm movements substantially differed between microgravity and normogravity. Our data therefore highlight the capacity of the central nervous system to perform very fast neuromuscular adjustments that are adapted to the gravitational constraints. We discuss our findings by considering the contribution of feedforward and feedback mechanisms in the online control of arm pointing movements.

## Introduction

On Earth, reaching movements performed by healthy human adults are usually precise and accurate in stable environments. Nevertheless, it is also puzzling to see that movements can remain accurate when unexpected changes occur during their execution (Won and Hogan, 1995). This is often the case for instance when we reach for an object that suddenly drops or moves from its initial location after movement initiation. This ability, referred to as sensorimotor flexibility (see Gomi, 2008 for a review), has been mainly investigated using the double-step paradigm. In the most used form of this paradigm, participants must correct their reaching trajectory when an unexpected target jump occurs after movement onset (Soechting and Lacquaniti, 1983; van Sonderen et al., 1989). By manipulating the context of double-step occurrence, previous studies showed that the rapid corrections applied to the ongoing movement are quite robust and efficient in the extent of reasonable spatio-temporal constraints (Goodale et al., 1986; Pélisson et al., 1986; Prablanc and Martin, 1992; Boulinguez et al., 2001; Sarlegna et al., 2003; Fautrelle et al., 2010, 2011; Oostwoud Wijdenes et al., 2011; Archambault et al., 2015). The question arises as to how sensorimotor flexibility is altered when the surrounding force field is substantially changed. For instance, in a weightless environment, can humans correct their ongoing movements if their spatial goal is suddenly changed after motor initiation?

Successful online motor corrections in terrestrial environments have been explained by the existence of a feedback controller allowing the central nervous system (CNS) to quickly process afferent information to bridge the gap between actual and intended arm spatial location (Carlton, 1981). In this framework, such a feedback controller would necessarily operate on a pre-established motor command elaborated by the CNS on the basis of initial state estimates about the body and world dynamics (i.e., feedforward control; Sabes, 2000). Remarkably, however, there is no consensus in the literature about the link between feedback and feedforward control of reaching or pointing movements. On the one hand, it has been suggested that feedback and feedforward controllers would act separately to adapt distinct motor features, respectively, related to either “*target errors*” or “*execution errors*” (Diedrichsen et al., 2005). Thus, while *target errors* could decrease through online corrective processes considering new behavioral goals, *execution errors* would require updating internal models (e.g., related to limb dynamics) to be reduced. In this vein, adaptive mechanisms to new force fields and online corrective processes may be considered, to a certain extent, as being independent. The existence of distinct neural correlates for feedback and feedforward controllers have been advanced to support this hypothesis. The posterior parietal cortex and the basal ganglia would play a key role in response to *target errors* (Desmurget et al., 1999, 2004), while the cerebellum and the motor cortex would be involved in the processes related to *execution errors* (Paz et al., 2003; Smith and Shadmehr, 2005). Alternatively, other works have led to the hypothesis that feedback and feedforward controllers are closely linked if not intertwined, particularly when considering adaptive processes. Recent studies, indeed, suggested that feedback-related mechanisms would directly use and implement new dynamics of the environment and body limbs in fast corrective responses (Kurtzer et al., 2008; Wagner and Smith, 2008; Cluff and Scott, 2013; Crevecoeur and Scott, 2014). In this case, a smart feedback controller, capable of rapidly updating sensory predictions based on short feedback latencies could continuously operate during motor control and learning (Crevecoeur et al., 2012, 2020).

The present study aims to challenge these two hypotheses by assessing human capabilities to produce online motor corrections consecutive to an unpredictable change of target location in microgravity. We recently reported that individuals with no experience in microgravity were able to execute accurate whole-body reaching at the earliest time of microgravity exposure during parabolic flights (Macaluso et al., 2017). This was presumably achieved by rapidly updating internal models from the new dynamic properties of the environment as suggested in previous studies (Papaxanthis et al., 2005; White et al., 2018), although gravity-independent factors, such as intersegmental dynamics may also be at work in updating the representation of limb dynamics (Flanders and Herrmann, 1992). The fact that movements performed in microgravity also exhibit kinematic features that mostly remained in the normogravity standards (e.g., Macaluso et al., 2017) supports an optimal reorganization of movement planning in a feedforward manner based on initial state estimates of the sensorimotor system. If the feedback controller ruling online motor corrections is linked to the -fast adapted- feedforward controller, we should also observe early accurate corrective responses to vertical target jumps in microgravity, preserving normogravity-like kinematics during initial microgravity exposure.

Alternatively, if the feedback controller works independently from the feedforward controller and therefore needs more time to adapt, then reaching errors in the upward direction (with possible higher peak velocities) should be observed in 0g, at least in the first trials with target jump because the arm unweighting would not be accounted for during movement corrections. In this case, several movement repetitions should be necessary to adjust the online corrective mechanisms according to the new force field requirements.

## Materials and Methods

### Participants

Seven right-handed adults (3 women and 4 men, mean age = 40 ± 6.5 years) participated in the experiment on a voluntary basis. None of the participants suffered from neuromuscular or sensory impairments, as confirmed by medical examination prior to the experiment. Before the parabolic flight, the participants were given comfort medication (scopolamine) to avoid motion sickness. This medication has been shown not to alter sensorimotor control (e.g., reflex circuitry and muscular activation and coordination; Ritzmann et al., 2016). All the participants were naive as to the specific purpose of the study, which was authorized by the ANSM (French National Agency for Biomedical Security) and approved by the related local ethics committee (CPP Sud Méditerranée – Agreement number 1604). The participants gave their signed informed consent prior to the study in accordance with the Helsinki Convention.

### Experimental Setup

**FIGURE 1:**
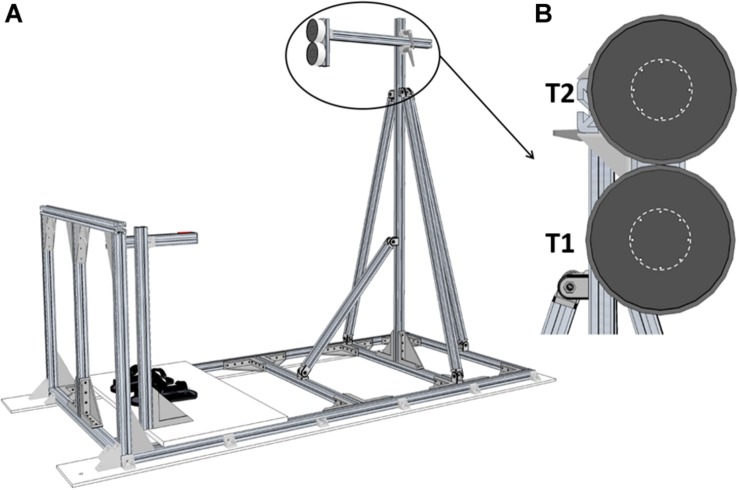
Experimental setup. **(A)** Global view of the pointing structure including targets, start push-button and footstraps. **(B)** Front view of the two targets. T1 and T2 were separated by 10 cm. The dotted line represents the area to be reached (Ø 4 cm).

The experiment was conducted onboard the A-310 ZERO-G aircraft chartered by the French national space research center (CNES) and Novespace during the CNES #128 parabolic flight campaign including 3 days of flight. Parabolic maneuvers were similar to those that we described in previous studies (Bringoux et al., 2012; Saradjian et al., 2013; Macaluso et al., 2017). Participants stood upright in front of two LEDs-defined circular targets (target area diameter: 4 cm) with their feet fastened to the cabin floor by means of footstraps (Figure 1A). The targets location was set according to the participants’ anthropometry: the first target (T1) was positioned in front of the right arm, at shoulder height and at arm length distance. The second target (T2) was positioned 10 cm above T1 (i.e., approximately at eye level; Figure 1B).

A push-button located on the right side of the participants was used to standardize the index finger starting position and to trigger target jump. It was adjusted to the participants’ height so that their right arm was kept outstretched alongside their body. Motion of the right arm was recorded using two infra-red active markers positioned onto the participants’ right index and shoulder which were recorded at 200 Hz by an optoelectronic motion capture system (Codamotion CXS and Active CodaHub, Charnwood Dynamics Ltd., Leicestershire, United Kingdom). Trials were elicited by switching T1 or T2 on. Target jumps were produced by switching T1 off and synchronously switching T2 on when the participants released the start push-button (sample frequency: 200 Hz).

### Procedure

All participants were tested in both normogravity (1g) and microgravity (0g) environments in two separate experimental sessions. Serving as a ground baseline condition, the 1g session was performed in the plane before the flight. The 0g environment corresponded to the successive weightless episodes during parabolic flight maneuver. Before each trial, participants stood upright with the arms outstretched along the body and the right index finger pressing the start push-button. Participants were instructed to initiate their movement as soon as one of the targets switched on. They had to point toward the target with their arm outstretched as fast as possible while primarily respecting accuracy constraints imposed by the size of the targets (i.e., accuracy over speed). Participants had to keep the final finger position until target extinction (3 s after movement onset) before returning to the starting position. In 20% of all trials (i.e., target jump trials), the initially illuminated T1 was switched off when the start push-button was released, while T2 was concomitantly switched on. In this double step condition, participants were asked to point toward the final illuminated target (T2) and avoid touching T1. They were informed before the experiment of the possible occurrence of target jumps but received no information regarding their rate and timing.

An experimental session was composed of 50 trials, including 20 single-step trials for each target (T1; T2), and 10 target jump trials (T_*jump*_). In 0g, these 50 trials were presented in 10 successive parabolas for each participant (i.e., 5 trials per parabola). The target conditions (T1; T2; T_*jump*_) were presented in a pseudorandom order which was counterbalanced between the participants. We ensured that the 3 target conditions occurred at least once among the five trials during each parabola. Each session lasted about 20 min.

### Data Processing

We replicated the analyses performed by Macaluso et al. (2017) who used a similar experimental setup in normo- and microgravity. Index finger and shoulder position data were filtered using a digital second-order dual-pass Butterworth filter (cutoff frequency 10 Hz). We analyzed the success rate (% of trials in which the index finger touched the target area), the finger endpoint deviation (absolute distance between index fingertip and target center along the Z vertical axis), reaction time (time elapsed between target illumination and movement onset), movement duration (time between movement onset and movement offset marked when the tangential velocity, respectively, reached above and dropped below 1.5% of its peak) and mean tangential velocity of the finger (*V*_*mean*_). Finger position data was differentiated to obtain the endpoint tangential velocity.

Using finger and shoulder data, we also computed the velocity and acceleration profiles of the arm angular elevation (i.e., the angle evolution of the extended arm around the shoulder with respect to its initial orientation). From the velocity profile, we extracted peak velocity (PV_*ang*_) and the relative angular deceleration duration (rDD_*ang*_). rDD_*ang*_ was defined as the duration between PV_*ang*_ and movement offset expressed in % of movement duration. The initial stage of motor execution was investigated by assessing peak acceleration (PA_*ang*_) and time to peak acceleration (TPA_*ang*_).

Repeated-measures analyses of variance (ANOVAs) were used for mean comparisons, following a two environment (1g; 0g) × 3 Target presentation (T1; T2; T_*jump*_) statistical design. Prior tests (Kolmogorov-Smirnov) confirmed the normality of the data. Newman-Keuls comparisons were used for *post-hoc* analyses and significance threshold was set at 0.05 for all statistical tests.

## Results

### Success Rate and Finger Endpoint Deviation

**FIGURE 2:**
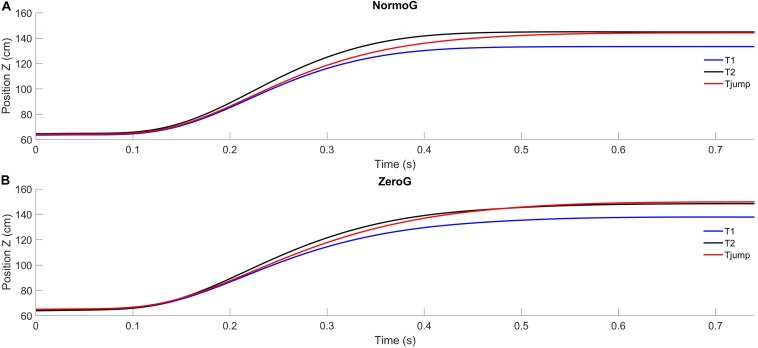
Average endpoint position (index fingertip) as a function of time in the XZ sagittal plane for the three target presentations in in normogravity (NormoG – Panel **A**) and microgravity (ZeroG – Panel **B**).

Mean arm trajectories obtained for T1, T2, and T_*jump*_ trials are illustrated in Figure 2. Overall, in both environments, pointing to unpredictable target jumps was performed successfully. Indeed, success rate in T_*jump*_ condition was 93.3% ± 13.3 in 1g and 90.5% ± 10.0 in 0g. The ANOVA performed on success rate revealed no significant main effect of the Environment (*F*_(1, 6)_ = 4.99; *p* = 0.07), no effect of Target presentation (although a trend may be found for a lower success rate in T_*jump*_ condition whatever the Environment; *F*_(2, 12)_ = 3.61; *p* = 0.06) and no significant interaction between these two factors (*F*_(2, 12)_ = 0,89; *p* = 0.44). Moreover, the analyses conducted on the successful trials revealed no main effect of the experimental conditions and no interaction between these factors on the finger endpoint deviation [Environment: *F*_(1, 6)_ = 2.96; *p* = 0.14; Target presentation: *F*_(2, 12)_ = 0.91; *p* = 0.43; Environment × Target presentation: *F*_(2, 12)_ = 1.36; *p* = 0.30].

### Reaction Time, Movement Duration, and Mean Tangential Velocity (Vmean)

Although close to significance, the ANOVA conducted on reaction time (mean = 294 ms ± 40) revealed no main effect of the Environment (*F*_(1, 6)_ = 5.96; *p* = 0.06). Moreover, no significant effect of Target presentation (*F*_(2, 12)_ = 0.56; *p* = 0.58) and no significant interaction between both factors were found (*F*_(2, 12)_ = 0.09; *p* = 0.92).

The ANOVA conducted on movement duration revealed a significant main effect of Target presentation (*F*_(2,12)_ = 5.53; *p* = 0.02). Movement duration in T_*jump*_ condition (596 ms ± 10) was longer than in T1 condition (559 ms ± 0.10; *p* = 0.02) but did not differ from T2 condition (577 ms ± 11; *p* = 0.13). No other significant main effect or interaction between both factors were found (Environment: *F*_(1, 6)_ = 4.59; *p* = 0.08; Environment × Target presentation: *F*_(2, 12)_ = 2.97; *p* = 0.09).

**FIGURE 3:**
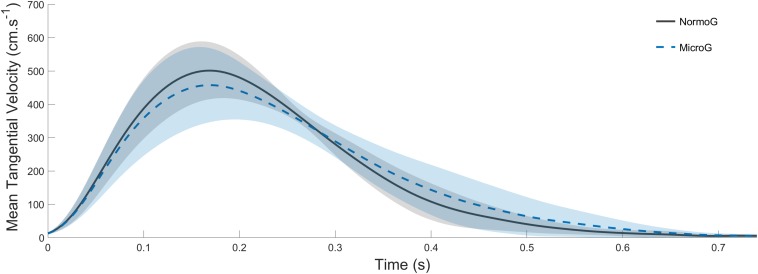
Average endpoint tangential velocity recorded in T_*jump*_ trials in both normo- (solid line) and microgravity (dotted line). Shaded areas represent between-subject standard deviation.

The ANOVA conducted on Vmean (mean = 294 cm.s^–1^ ± 40; Figure 3) revealed no main effect of the Environment (*F*_(1,6)_ = 4.88; *p* = 0.07) and no main effect of Target presentation (*F*_(2, 12)_ = 3.41; *p* = 0.07). Moreover, no significant interaction between both factors were found (*F*_(2, 12)_ = 2.42; *p* = 0.13).

To sum up, microgravity did not significantly affect pointing performance, even when unpredictable target jumps occurred at movement onset. In this unusual environment, there was no substantial disruption of the general spatiotemporal characteristics of the movements. Then, we investigated the effect of gravitational constraints on upper limb movements by assessing the kinematics of arm pointing movements.

### Peak Angular Velocity (PV_*ang*_) and Relative Angular Deceleration Duration (rDD_*ang*_)

**FIGURE 4:**
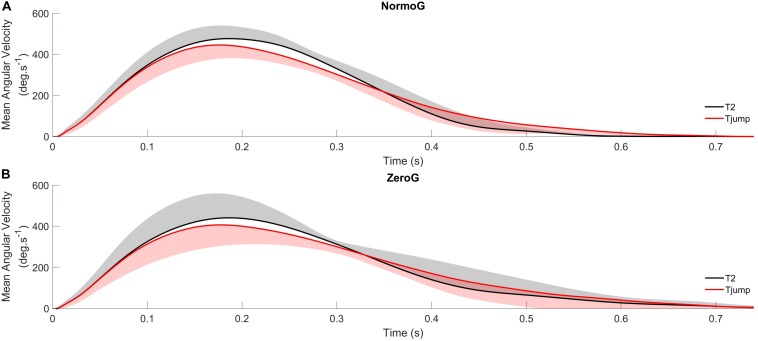
Average angular velocity profiles of arm pointing for T_*jump*_ and T2 in normogravity (NormoG – Panel **A**) and microgravity (ZeroG – Panel **B**), illustrating the distinct kinematics between both target.

The ANOVA conducted on PV_*ang*_ revealed a significant main effect of Target presentation (*F*_(2,12)_ = 51.15; *p* < 0.001). PV_*ang*_ in T2 condition (471 deg.s^–1^ ± 87) was greater than in T1 (439 deg.s^–1^ ± 81; *p* < 0.001) and T_*jump*_ (437 deg.s^–1^ ± 84; *p* < 0.001; Figure 4) conditions while PV_*ang*_ did not significantly differ between T1 and T_*jump*_ conditions (*p* = 0.69). No other significant main effect or interaction between both factors were found (Environment: *F*_(1, 6)_ = 1.18; *p* = 0.32; Environment × Target presentation: *F*_(2, 12)_ = 0.56; *p* = 0.59).

**FIGURE 5:**
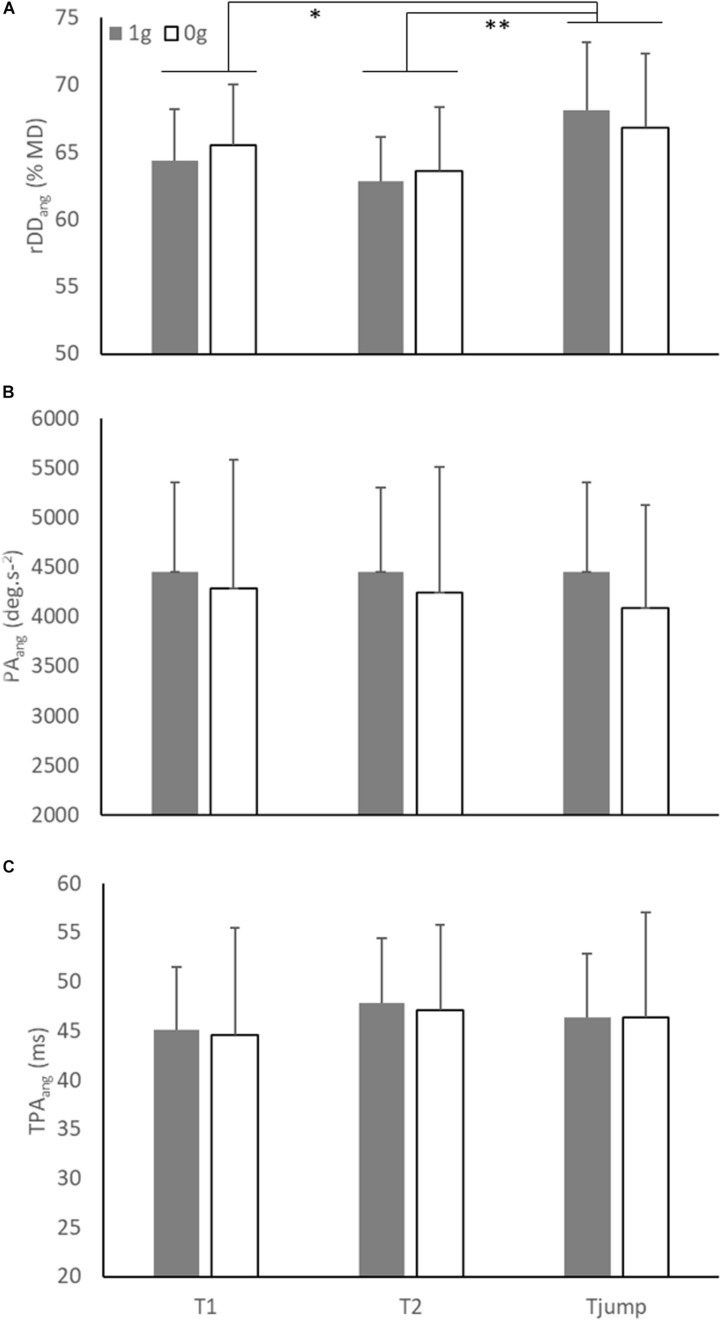
**(A)** Mean relative angular deceleration duration (rDD_*ang*_), **(B)** mean peak angular acceleration (PA_*ang*_), and **(C)** mean time to peak angular acceleration (TPA_*ang*_) as a function of environment and target presentation. Error bars represent 95% confidence interval. Significant differences were noted by stars (* = *p* < 0.05; ** = *p* < 0.01).

Similarly, the ANOVA conducted on rDD_*ang*_ showed a significant main effect of Target presentation (*F*_(2,12)_ = 10.90; *p* = 0.002). rDD_*ang*_ in T_*jump*_ condition (67.5 % ± 5.6) was greater than in T1 (64.9 % ± 4.4; *p* = 0.02) and T2 (63.2 % ± 4.3; *p* = 0.002; Figure 4) conditions, while rDD_*ang*_ was not significantly different between T1 and T2 conditions (*p* = 0.08). However, as illustrated in Figure 5A, no significant main effect of the Environment and no interaction between both factors were found (Environment: *F*_(1, 6)_ = 0.06; *p* = 0.81; Environment × Target presentation: *F*_(2, 12)_ = 2.20; *p* = 0.15).

### Peak Angular Acceleration (PA_*ang*_) and Time to Peak Angular Acceleration (TPA_*ang*_)

As shown in Figure 5B, the ANOVA conducted on PA_*ang*_ (mean = 4332 deg.s^–2^ ± 1080) did not reveal significant main effect of Environment (*F*_(1, 6)_ = 0.56; *p* = 0.48) and Target presentation (*F*_(2, 12)_ = 1.23; *p* = 0.33) nor significant interaction between both factors (*F*_(2, 12)_ = 1.11; *p* = 0.36).

Similarly, as illustrated in Figure 5C, the ANOVA conducted on TPA_*ang*_ (mean = 46 ms ± 9) showed no significant effect of Environment (*F*_(1, 6)_ = 0.02; *p* = 0.88), Target presentation (*F*_(2, 12)_ = 1.21; *p* = 0.33) and no significant interaction between both factors (*F*_(2, 12)_ = 0.02; *p* = 0.98).

### Stability of T_*jump*_ Performance in 0g

**FIGURE 6:**
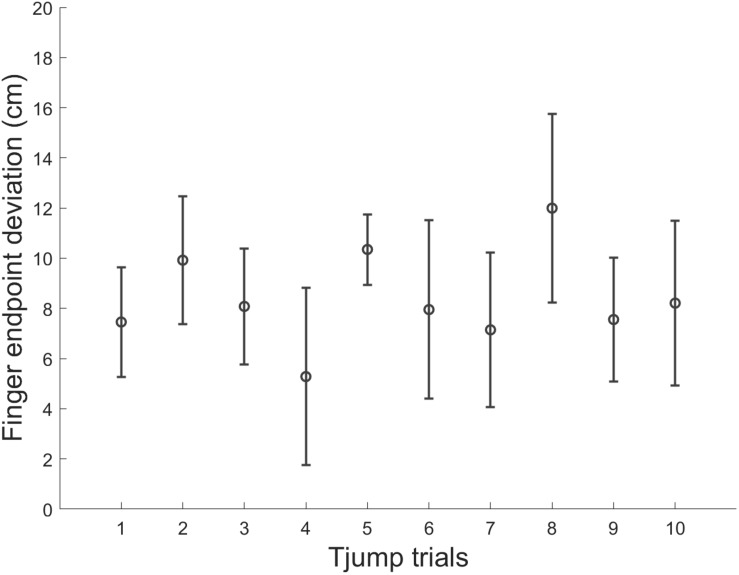
Mean finger endpoint deviations in each T_*jump*_ trial along the experimental session in microgravity. Errors bars represent between-subject standard deviation.

Finally, we assessed the potential influence of adaptation to target jumps in microgravity throughout the parabolic flight. We compared for each variable mentioned above, T_*jump*_ trials performed during the 1st, 5th, and 10th parabola (i.e., 1 trial per parabola) using one way repeated-measures ANOVAs. None of the variable was significantly affected by parabola number (*p* > 0.05). Thus, motor responses to target jump in microgravity did not significantly vary throughout the experiment (Table 1 and Figure 6).

**TABLE 1:** Mean individual values and intrasbject standard deviation (SD) for the angular deceleration duration relative to movement time (rDD_*ang*_), the peak angular acceleration (PA_*ang*_) and time to peak angular deceleration (TPA_*ang*_) as a function of the environment (1g vs 0g).

	rDDang				PAang				TPAang			
			
	1g		0g		1g		0g		lg		0g	
						
Participant	Mean (%)	SD	Mean (%)	SD	Mean	SD	Mean	SD	Mean	SD	Mean	SD
P1	62.3	4,6	66.5	4,4	3521 deg/s^2^	703	3010 deg/s^2^	389	41.9 ms	9,6	26.7 ms	2,5
P2	72.2	3,0	71.5	2,9	3260 deg/s^2^	428	3284 deg/s^2^	546	42.8 ms	7,5	45.0 ms	25,7
P3	71.5	8,0	67.6	7,9	5664 deg/s^2^	720	4890 deg/s^2^	933	36.7 ms	3,5	50.0 ms	24,7
P4	65.9	7,0	61.5	5,5	5558 deg/s^2^	751	5365 deg/s^2^	818	47.5 ms	7,1	66.0 ms	18,4
P5	72.6	5,1	72.9	2,5	4099 deg/s^2^	592	5393 deg/s^2^	654	45.5 ms	5,5	43.0 ms	4,2
P6	59.5	2,5	56.4	5,6	5095 deg/s^2^	1064	3937 deg/s^2^	479	53.5 ms	11,6	47.5 ms	10,4
P7	72.7	3,5	71.3	6,0	3991 deg/s^2^	537	2729 deg/s^2^	359	57.0 ms	6,7	46.1 ms	12,2
All participants	68.1	4,8	66.8	5,0	4455 deg/s^2^	685	4087 deg/s^2^	597	46.4 ms	7,4	46.3 ms	14,0

In summary, the overall pointing performance and the kinematics of arm angular elevation were unaffected in microgravity, even in presence of target jumps. Kinematics of arm elevation, however, depended on the target condition. Direct pointing movements toward the upper target (T2) exhibited greater peak velocity than those directed toward the lower target (T1) or toward the upper target following target jump (T_*jump*_). In this latter condition, the relative deceleration duration significantly increased as compared to pointing movements performed toward the stationary T1 or T2 targets. However, these changes were not dependent from the gravitational environment and were not the consequence of an adaptive process, as attested by the absence of significant variations in performance between the 1st the 5th, and the 10th parabolas during 0g exposure.

**FIGURE 7:**
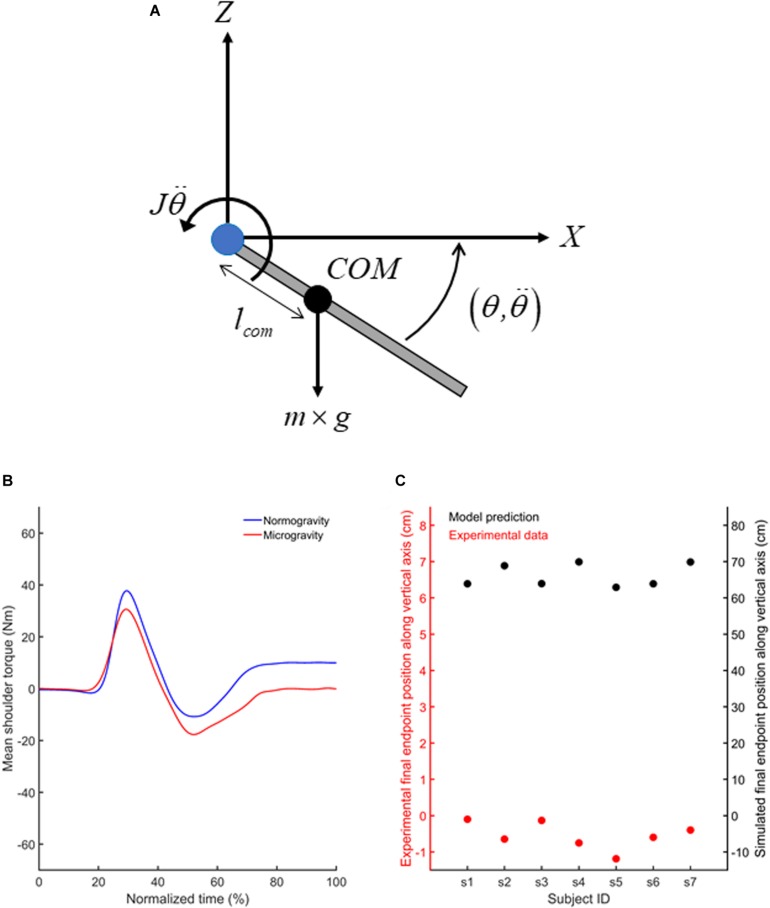
**(A)** Free body diagram of the extended upper limb showing the location of the upper limb center of mass (COM; black circle) and the lever arm (*l*_com_) of the COM with respect to the shoulder joint (blue circle). Also illustrated are the gravitational force (*m*×*g*) acting at the center of mass of the upper limb and the inertial torque (w). Summation of torques with respect to the shoulder joint leads to Eq. 1 provided in the text. **(B)** Mean shoulder torques computed using the inverse dynamical model for movement directed toward T1 in normogravity and microgravity. **(C)** Mean vertical finger endpoint position shown by each participant (red circles) when reaching toward T1 in microgravity (left y axis) and endpoint positions predicted by the forward dynamic model (black circles) if the participants would have used a 1 g internal model to produce their movements in microgravity (right *y* axis). Similar results (i.e., shoulder torques and vertical finger endpoint positions) were observed for movements directed toward T2 (not shown).

Together, our results suggest that the adaptation of feedforward mechanisms to the microgravity environment enabled feedback control mechanisms to operate efficiently from the first occurrence of a target jump. Importantly, the assumption that feedforward mechanisms were adapted to microgravity implies that different arm motor commands were produced to reach the targets in normo- and microgravity. To test this assumption, we used the laws of physics (i.e.,Newton–Euler equations) to estimate the shoulder torque using an inverse dynamics equation of motion for a two-dimensional movement (vertical plane, Figure 7A) of one-joint planar link-segment model (Otten, 2003). We evaluated the change in shoulder torques when the participants produced their vertical upper limb movements in microgravity relative to when the movements were performed in normogravity. To estimate shoulder torque (*T*_*s*_) in microgravity, gravitational acceleration (*g*) was equalled to zero in Eq. 1. Anthropometrical data of each participant permitted to calculate the mass of the upper limb (*m*), its moment of inertia (*J*) and the center of mass position (*l*_com_) with respect to shoulder joint (Winter, 2009). Using the upper limb angular acceleration $\left(\ddot{\theta }\right)$ and angular position (θ) when participants reached to T1 and T2 targets, we calculated for each participant and target, the net $\left(J\,\ddot{\theta }\right)$ and gravitational (*l*_com_×*m*×*g*×sin(−90−θ)) torques, respectively (Figure 7A).

(1) $$T_{s}=J\,\ddot{\theta }-\left(l_{\text{com}}\times m\times g\times \sin\,\left(-90-\theta \right)\right)$$

The outcomes of the inverse dynamic model confirmed that participants used different motor commands to reach the targets in microgravity and normogravity. Compared to normogravity, movements produced in microgravity showed smaller positive torque during arm acceleration and greater negative torque during arm deceleration (Figure 7B). These changes in shoulder torques indicate that in the absence of gravity, smaller and greater motor commands were required to accelerate and decelerate the vertical arm movements, respectively. In normogravity, after movement offset, a constant positive shoulder torque counterbalanced the gravitational torque to keep the arm still. In microgravity, the shoulder torque remained close to zero after the movements was performed.

We also compared the finger vertical endpoint recorded in microgravity with the prediction of the vertical endpoints made by the forward dynamic model if the participants would have used a 1g internal model to produce their movements in microgravity. To predict upper limb angular acceleration $\left(\overset{⌢}{\ddot{\theta }}\right)$ if the participants would have included the gravitational torque within their motor commands in microgravity, we used forward dynamics (Eq. 2).

(2) $${\overset{⌢}{\ddot{\theta }}}_{\text{microgravity}}=\frac{T_{\text{normogravity}}}{J}$$

From the double integration of the predicted angular acceleration, we predicted the angular position $\left(\overset{⌢}{\theta }\right)$ and calculated the predicted final index position along the vertical axis ${\overset{⌢}{Z}}_{\text{index}}$by calculating the vertical position of the index (Eq. 3; *l*_*upperlimb*_ represents the length between shoulder joint and index). Note that angular position during upper limb elevation was limited to 88° in accord with the normative range of motion of the glenohumeral joint of our participants (Stubbs et al., 1993).

(3) $${\overset{⌢}{Z}}_{\text{index}}=l_{\text{upperlimb}}\times \text{sin}\left(\overset{⌢}{\theta }\right)$$

Prediction of the forward dynamic model clearly demonstrated that participants needed to adapt their motor commands in microgravity to reach the targets (Figure 7C). Indeed, paired *t*-tests indicated that the vertical final endpoint position observed in microgravity were significantly lower than the final endpoint position that would have been observed if the participants would have used the same shoulder torque as in normogravity (empirical data vs biomechanical model, T1: [*t*(7) = 59.99, *p* < 0.001); T2: (*t*(7) = 61.87, *p* < 0.001)].

## Discussion

The present study aimed to challenge the sensorimotor flexibility of fast and accurate goal-directed pointing movements performed in microgravity which have been observed in previous studies (e.g., Papaxanthis et al., 2005; Macaluso et al., 2017; White et al., 2018) by presenting unpredictable jumps in target location following movement onset. Our results revealed that the overall performance and kinematics of motor corrections in response to target jumps were unaffected during the 0g phases of parabolic flight as compared to 1g baseline. These observations are remarkable when considering the substantial change in shoulder torques produced by the participants for accelerating and decelerating their movements in microgravity (Figure 6). We will review the main outcomes from these findings, first at a behavioral level and then, at a conceptual level referring to motor control and adaptation processes.

### Preserved Sensorimotor Flexibility in Microgravity

In response to upward target jumps that occurred at arm pointing initiation, participants were able to rapidly correct their ongoing movements as when they directly reached the upper target. Indeed, success rate, endpoint accuracy, reaction time and movement duration were not significantly different in T2 and T_*jump*_ conditions. The only motor changes observed between these conditions were a decrease of peak velocity and a lengthening of deceleration duration for pointing movements performed in T_*jump*_ condition compared to those performed in T2 condition (Figure 4). Likely, reducing the peak velocity allowed appropriate corrective mechanisms to occur through visuomotor feedback loops during the deceleration phase without additional processing time (Komilis et al., 1993). Consistent with this interpretation, several studies have reported immediate online corrective mechanisms following changes in target location which were not detrimental to the overall duration and accuracy of pointing (Gomi, 2008; Gaveau et al., 2014; for reviews).

The key finding of the present experiment is that the characteristics of online movement corrections were unaffected by the gravitational context, that is, pointing kinematics following target jump remained comparable in normo- and microgravity (Figures 3 and 5). Although a trend for significance was found for a lower success rate and a longer reaction time in 0g, the kinematic variables that are typically used for investigating online corrections, such as peak velocity, relative deceleration duration, peak acceleration and time to peak acceleration did not differ between both environments. Most importantly, these characteristics were effective without substantial exposure or trial repetition (i.e., the behavior remaining stable throughout the exposure to microgravity).

While pioneer experiments on pointing movements in microgravity reported noticeable changes in terms of speed or accuracy with respect to land observations (Berger et al., 1997; Mechtcheriakov et al., 2002), they also reported that these movements were influenced by task and environment constraints (Bock, 1998). As such, movement velocity requirements and target properties (e.g., size, position, number) are examples of parameters likely influencing motor planning and online correction efficiency. By considering these constraints, recent findings suggested fast and optimal adaptation of goal-directed movements in microgravity when the targets remain stationary (Gaveau et al., 2016; Macaluso et al., 2017). For instance, we observed readily adapted upward arm pointing in 0g with preserved accuracy and movement duration as compared to 1g observations (Macaluso et al., 2017). These adapted movements were characterized by immediate changes in early kinematics, likely due to a reorganization of motor planning based on initial state estimates (Rousseau et al., 2016). Specifically, and contrasting with the present findings, we reported in Macaluso et al. (2017) that single step pointing movements performed in 0g exhibited a greater peak acceleration and a longer relative deceleration duration compared to those performed in 1g. The discrepancy between our previous results and those of the present study could be explained by the presence of trials with and without target jumps. Indeed, it has been shown that target uncertainty can interfere with sensorimotor control and online motor corrections (Acerbi et al., 2017). According to the optimal feedback control theory (Todorov and Jordan, 2002; Scott, 2004), risk sensitivity (here inherent to the possibility of missing the target when it is unpredictably moved) is considered in the cost function to determine optimal motor output (Nagengast et al., 2010). In the present experiment, we can thus hypothesize that the CNS adopted a conservative motor strategy (e.g., by lengthening the relative deceleration phase) enabling the participants to keep the accuracy requirements while being alert to react to target jump. Our findings remarkably demonstrated that this motor strategy remained unchanged in microgravity and was fully adapted to the constraints of this unusual environment. The comparable online corrective responses in both 0g and 1g environments therefore provide evidence that the sensorimotor flexibility is preserved in 0g. The following section will focus on the possible outcomes that can be drawn from these behavioral findings onto the knowledge of feedforward-feedback adaptive mechanisms.

### On the Link Between Feedforward and Feedback Controllers

One of the main objectives of the present study was to assess the relationship between feedforward and feedback controllers governing motor production in altered gravitational fields. Our data are consistent with an imbrication of both controllers, considering the early-adapted corrective loops following target jump in microgravity.

Based on our previous findings (Bringoux et al., 2012; Macaluso et al., 2017), initial state estimates of the moving limb dynamics in altered gravity appear adequate to adapt the feedforward controller for successful reaching. Updating such state estimates for motor planning could be achieved in the early phase of exposure, even prior to pointing execution, based on torque-related proprioceptive information (Bringoux et al., 2012; Rousseau et al., 2016). The vestibular system could also have a key role in this updating process as it provides valuable information for estimating the gravitoinertial force field and for predicting its effects on the moving arm (Bockisch and Haslwanter, 2007; Guillaud et al., 2011; see Blouin et al., 2015 for a review). Here, by showing non-degraded pointing toward both vertical targets and corresponding 1g-like movement kinematics in microgravity, we confirmed that the feedforward controller could be rapidly adapted in response to the task and environment constraints.

Most importantly, the present study provides clear evidence for a coincident updating of the feedback controller in microgravity, as online motor corrections following unpredicted target jumps were effective and adapted without significant changes across trials. Early sensorimotor adaptation enabled arm pointing following target jump to remain unchanged with respect to normogravity standards regarding its spatiotemporal organization. Noticeably, keeping the same kinematics in different gravitational environments implies changing muscle activation. This was confirmed by our biomechanical model showing that shoulder torques markedly differed between normo- and microgravity (Figure 7).

The question arises as to whether the concurrent adaptive processes of feedforward and feedback controllers operate in parallel as the result of independent mechanisms (Diedrichsen et al., 2005) or within a nested architecture (Kurtzer et al., 2008). Consistent with the latter hypothesis, it has been demonstrated that adaptation to visuomotor rotation influences online movement correction when reaching for a target that suddenly changes position (Hayashi et al., 2016). Wagner and Smith (2008) and more recently Cluff and Scott (2013) also reported updated corrective mechanisms as soon as the feedforward controller has been adapted to a novel force field. Specifically, these authors showed that the learned dynamics of a velocity-dependent force field acting upon the moving limb directly transferred to the feedback-based online responses following unanticipated pulse perturbation produced by a robot manipulandum at the effector level. Based on their observations, the authors argued for the existence of a “smart feedback controller” that automatically scaled to changes in the internal models of limb dynamics. This is also in line with recent findings from Crevecoeur et al. (2020), suggesting a real-time learning algorithm as a potential link between online control and trial-to-trial adaptation for reaching movements in novel force fields. Trial-to-trial changes expressed during feedforward adaptation may also reflect changes in (model-free) online control strategies (Crevecoeur et al., 2019). Here, we extent this interpretation to visually mediated perturbations in altered gravitational contexts acting on body dynamics. Our findings suggest that feedforward modifications induced by 0g exposure are expressed in feedback control policy for handling a change in goal-directed specification (i.e., target jump).

## Conclusion

To our knowledge, the present study is the first to show successful -early adapted- corrections of ongoing arm pointing movements following unpredictable target jumps in microgravity. Our data provide new evidence for unimpaired sensorimotor flexibility in altered gravity, presumably derived from rapid updating of internal models used for motor planning. Overall, these findings shed new lights on human motor behavior and the possible link between feedforward and feedback controllers. These observations must be framed within the present experimental context which included upward target jumps of small magnitude and single joint pointing responses. Also, we cannot exclude more subtle or progressive adaptations to longer exposure to microgravity such as during long-term space missions (Reschke et al., 2002). Further research is needed to clarify how these adaptive mechanisms arise at muscular and neurophysiological levels. EMG and EEG recordings would be valuable to investigate how the CNS keeps the kinematics of online corrections comparable between different force fields, hence addressing the issue of optimality in the control of pointing movements (Berret et al., 2008; Gaveau et al., 2016).

## Data Availability Statement

The datasets generated for this study are available on request to the corresponding author.

## Ethics Statement

The study was authorized by the ANSM (French National Agency for Biomedical Security : Authorization n° 15 014) and approved by the related local ethics committee (CPP Sud Méditerranée– Agreement number 1604). The participants gave their signed informed consent prior to the study in accordance with the Helsinki Convention and the CPP agreement.

## Author Contributions

LB designed and performed the experiment, analyzed the data and wrote the manuscript. TM designed and performed the experiment, analyzed the data and reviewed the manuscript. PS designed and performed the experiment and reviewed the manuscript. LC analyzed the data and reviewed the manuscript. FB designed and performed the experiment, and reviewed the manuscript. LM designed the experiment and reviewed the manuscript. MS developed the biomechanical model and reviewed the manuscript. JB designed the experiment and reviewed the manuscript.

## Conflict of Interest

The authors declare that the research was conducted in the absence of any commercial or financial relationships that could be construed as a potential conflict of interest.
